# Assessment of diastolic function from velocity-encoded cardiac magnetic resonance data in patients with hypertrophic cardiomyopathy

**DOI:** 10.1186/1532-429X-15-S1-P170

**Published:** 2013-01-30

**Authors:** Golmehr Ashrafpoor, Nadjia Kachenoura, Emilie Bollache, Laurent Macron, Arshid Azarine, Eric Bruguière, Sébastien Fontaine, Michel Desnos, Albert A Hagège, Elie Mousseaux, Alban Redheuil

**Affiliations:** 1 Hôpital Européen Georges Pompidou, Paris, France; 2 Inserm U678, Université Pierre et Marie Curie Paris 6, Paris, France

## Background

Diastolic dysfunction evaluation may be relevant for early diagnosis of hypertrophic cardiomyopathy (HCM) and subsequent risk assessment. The aim of our study was to assess phase contrast cardiac magnetic resonance (PC-CMR) diastolic parameters obtained with a semi-automated method in relation to left ventricular (LV) remodeling and late gadolinium enhancement (LGE) in patients with HCM.

## Methods

We studied 48 patients with HCM and 23 healthy volunteers matched for age, sex and body surface area (BSA). Mitral inflow and myocardial velocities were assessed using through plane 2D PC-CMR (VENC=150cm/s and 20cm/s respectively). Transmitral peak flow-rates (Ef and Af) and early E' peak myocardial longitudinal velocity were obtained semi-automatically using CardFlow (INSERM U678). LV volumes, segmental thickness and mass were obtained from SSFP images. LGE volume was quantified semi-automatically using a 6 SD threshold.

## Results

Peak myocardial longitudinal velocity E' was significantly lower and E/E' was significantly higher in patients with HCM compared with controls (Table). We found a linear relationship between decreased E' and increased LV mass index (p<0.0001), decreased mass/end-diastolic volume (M/EDV) ratio (p<0.0001), increased LGE mass (p=0.04) and LGE extension (p=0.04). The relationships between E' and LV mass index and M/EDV were independent of age, gender, BSA and systolic blood pressure (p<0.001).

**Table 1:** 

	Controls (n=23)	HCM (n=48)	p
LV mass (g)	132 (33)	195 (69)	0.0001
LV mass index (g/m2)	69 (13)	106 (36)	<0.0001
EDV (ml)	139 (37)	151 (45)	NS
ESV (ml)	50 (14)	51 (23)	NS
Mass/EDV (g/ml)	0.99 (0.3)	1.35 (0.5)	0.0026
LV ejection fraction (%)	64 (5)	66 (10)	NS
Maximal wall thickness (mm)	NA	20 (4)	NA
Extent of LV hypertrophy (%)	NA	16 (17)	NA
LGE (g)	NA	4.1 (4.6)	NA
LGE (%)	NA	2 .3 (2.2)	NA
Ef/Af	1.21 (0.7)	1.47 (2.3)	NS
E' (cm/s)	8.6 (4.7)	3.4 (1.6)	<0.0001
E/E'	8.1 (4.3)	25.3 (18.6)	0.0001
DT (ms)	213.9 (72.1)	241.4 (67.4)	0.11

LV, left ventricular; EDV, end-diastolic volume; ESV, end-systolic volume; LGE, late gadolinium enhancement; DT, deceleration time

## Conclusions

Comparison of patients with HCM and healthy volunteers by CMR showed significantly altered LV diastolic function related to LV hypertrophy and LGE. Assessment of diastolic function may be considered for a comprehensive cardiac evaluation in HCM.

## Funding

None

